# Coronary Heart Disease-Associated Variation in *TCF21* Disrupts a miR-224 Binding Site and miRNA-Mediated Regulation

**DOI:** 10.1371/journal.pgen.1004263

**Published:** 2014-03-27

**Authors:** Clint L. Miller, Ulrike Haas, Roxanne Diaz, Nicholas J. Leeper, Ramendra K. Kundu, Bhagat Patlolla, Themistocles L. Assimes, Frank J. Kaiser, Ljubica Perisic, Ulf Hedin, Lars Maegdefessel, Heribert Schunkert, Jeanette Erdmann, Thomas Quertermous, Georg Sczakiel

**Affiliations:** 1 Department of Medicine, Division of Cardiovascular Medicine, and Cardiovascular Institute, Stanford University School of Medicine, Stanford, California, United States of America; 2 Institut für Molekulare Medizin, Universität zu Lübeck, Lübeck, Germany; 3 Department of Medicine, Division of Cardiothoracic Surgery, and Cardiovascular Institute, Stanford University School of Medicine, Stanford, California, United States of America; 4 Institut für Humangenetik, Universität zu Lübeck, Lübeck, Germany; 5 DZHK (German Research Centre for Cardiovascular Research), partner site Hamburg/Lubeck/Kiel, Lubeck, Germany; 6 Center for Molecular Medicine, Karolinska Institutet, Stockholm, Sweden; 7 Deutsches Herzzentrum München, Technische Universität München, Munich, DZHK, partner site Munich Heart Alliance, Munich, Germany; 8 Institut für Integrative und Experimentelle Genomik, Universität zu Lübeck, Lübeck, Germany; University of Oxford, United Kingdom

## Abstract

Genome-wide association studies (GWAS) have identified chromosomal loci that affect risk of coronary heart disease (CHD) independent of classical risk factors. One such association signal has been identified at 6q23.2 in both Caucasians and East Asians. The lead CHD-associated polymorphism in this region, rs12190287, resides in the 3′ untranslated region (3′-UTR) of *TCF21*, a basic-helix-loop-helix transcription factor, and is predicted to alter the seed binding sequence for miR-224. Allelic imbalance studies in circulating leukocytes and human coronary artery smooth muscle cells (HCASMC) showed significant imbalance of the *TCF21* transcript that correlated with genotype at rs12190287, consistent with this variant contributing to allele-specific expression differences. 3′ UTR reporter gene transfection studies in HCASMC showed that the disease-associated C allele has reduced expression compared to the protective G allele. Kinetic analyses *in vitro* revealed faster RNA-RNA complex formation and greater binding of miR-224 with the *TCF21* C allelic transcript. In addition, *in vitro* probing with Pb^2+^ and RNase T1 revealed structural differences between the *TCF21* variants in proximity of the rs12190287 variant, which are predicted to provide greater access to the C allele for miR-224 binding. miR-224 and *TCF21* expression levels were anti-correlated in HCASMC, and miR-224 modulates the transcriptional response of *TCF21* to transforming growth factor-β (TGF-β) and platelet derived growth factor (PDGF) signaling in an allele-specific manner. Lastly, miR-224 and TCF21 were localized in human coronary artery lesions and anti-correlated during atherosclerosis. Together, these data suggest that miR-224 interaction with the *TCF21* transcript contributes to allelic imbalance of this gene, thus partly explaining the genetic risk for coronary heart disease associated at 6q23.2. These studies implicating rs12190287 in the miRNA-dependent regulation of *TCF21*, in conjunction with previous studies showing that this variant modulates transcriptional regulation through activator protein 1 (AP-1), suggests a unique bimodal level of complexity previously unreported for disease-associated variants.

## Author Summary

Both genetic and environmental factors cumulatively contribute to coronary heart disease risk in human populations. Large-scale meta-analyses of genome-wide association studies have now leveraged common genetic variation to identify multiple sites of disease susceptibility; however, the causal mechanisms for these associations largely remain elusive. One of these disease-associated variants, rs12190287, resides in the 3′untranslated region of the vascular developmental transcription factor, *TCF21*. Intriguingly, this variant is shown to disrupt the seed binding sequence for microRNA-224, and through altered RNA secondary structure and binding kinetics, leads to dysregulated *TCF21* gene expression in response to disease-relevant stimuli. Importantly *TCF21* and miR-224 expression levels were perturbed in human atherosclerotic lesions. Along with our previous reports on the transcriptional regulatory mechanisms altered by this variant, these studies shed new light on the complex heritable mechanisms of coronary heart disease risk that are amenable to therapeutic intervention.

## Introduction

Coronary heart disease (CHD), involving atherosclerosis and myocardial infarction (MI), is a genetically complex trait and represents the leading cause of mortality worldwide. Meta-analyses of genome-wide association studies (GWAS) for CHD have identified 46 replicated loci in subjects of European descent [1]. Of these loci, the region at 6q23.2 contains the lead variant, rs12190287, which had the lowest *P* value among several SNPs that reached the genome-wide significance threshold in this locus [2]. rs12190287 is located within an exon of the basic-helix-loop-helix transcription factor *TCF21*, and represents an expression quantitative trait locus (eQTL) for this gene by regulating *TCF21* gene expression levels in omental adipose and liver tissues [2]. Importantly, the *TCF21* locus association with CHD was recently confirmed in a meta-analysis of predominantly European subjects genotyped with the Cardio-Metabochip (Illumina) and in a three stage GWAS for CHD in individuals of Han Chinese descent [1], [3].

The association of *TCF21* with CHD is particularly compelling, given its association with fundamental cardiovascular embryonic events that might relate to subsequent responses to cardiovascular injury. Tcf21 has recently been shown to regulate cell-fate determination and stages of cell differentiation throughout coronary vascular development in mice. *Tcf21* was shown to mark populations of mesodermal-derived cells in the proepicardial organ (PEO) at embryonic day 9.5, and mesenchymal-derived cells in the developing pericardium at later time points [4]–[7]. Global knockout studies in mice have confirmed an important role for *Tcf21* in the formation of coronary artery smooth muscle cells and cardiac fibroblasts [8], [9]. *Tcf21* deletion results in aberrant smooth muscle cell (SMC) differentiation and an absence of cardiac fibroblasts, as evidenced by increased epicardial SMC marker expression [8]. Together, these mouse studies suggest that loss of *Tcf21* expression leads to SMC expansion while sustained expression is essential to cardiac fibroblast maturation, likely through regulation of multipotent precursor cell fate.

Recent work in this laboratory has identified a *cis*-acting mechanism by which the protective *TCF21* G allele at variant rs12190287 disrupts an activator protein 1 (AP-1)-like enhancer element, to alter allele specific transcriptional control of *TCF21* gene expression [10]. Interestingly, this cis-regulatory element modulates growth factor (platelet-derived growth factor receptor beta-β) and epicardial development (Wilms tumor 1) signaling pathways in coronary artery SMC [10]. In complementary studies reported herein we provide evidence that the 3′-untranslated region (3′-UTR) of *TCF21* binds miR-224 to regulate expression of this gene, and that this regulation is obviated by the minor allele which confers a seed mismatch to disrupt miR-224 binding and accessibility of this region of the *TCF21* 3′-UTR. To our knowledge, these data provide the first example of miRNA binding disruption as a likely mechanism for a CHD risk gene association, and the first example of concurrent miRNA and transcriptional regulation at a single disease associated causal variant.

## Results

### Allelic expression imbalance at rs12190287 in circulating leukocytes and HCASMC

**Figure 1:**
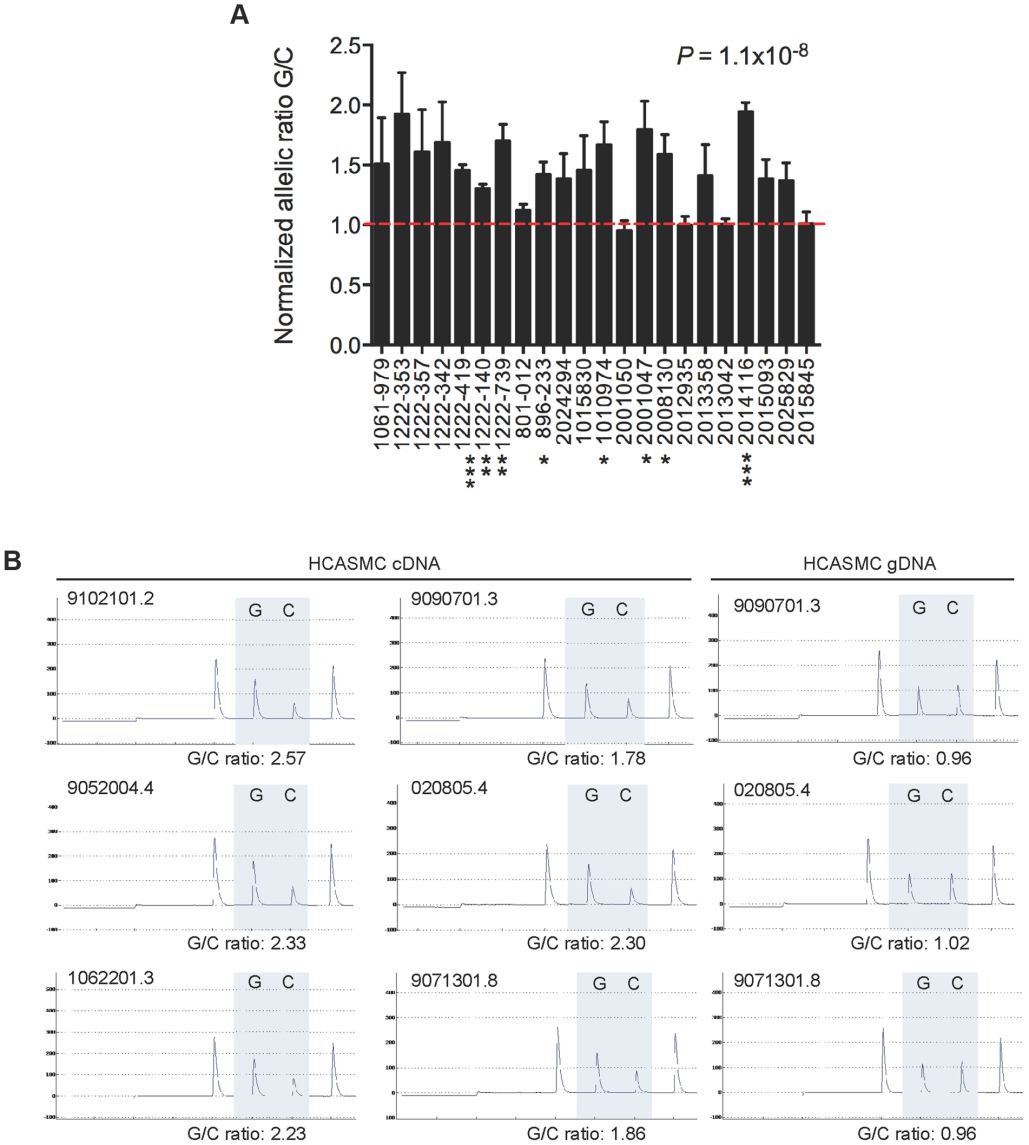
Allele specific expression at rs12190287 in human peripheral blood samples. (**A**) TaqMan quantitative PCR results depicting allele specific expression of *TCF21* at rs12190287 in peripheral blood samples (n = 22 heterozygous samples) obtained from human cohort studies. Allelic expression and genotyping data were determined from cDNA and gDNA, respectively, using a pre-calibrated TaqMan SNP genotyping probe for rs12190287, with each sample performed in triplicates. Data are expressed as the normalized allelic ratio of cDNA/gDNA and values represent mean ± SEM. (**B**) Representative pyrosequencing traces from HCASMC cDNA and gDNA from various cell lots. Allelic ratios were determined from the area under the curve for major and minor allele. Similar results were observed from three independent experiments. *P* values shown were calculated from combined data using a paired *t*-test compared to allelic ratio of gDNA samples. Asterisks represent individual level of significance versus expected allelic ratio of 1.0, * P<0.05, **P<0.01, ***P<0.001.

To better understand the mechanisms of disease risk at 6q23.2, we set out to define causal variation among the CHD-associated SNPs by examining the allele specific expression (ASE) in heterozygous individuals for the transcript variant rs12190287, which is located in the 3′-UTR of the *TCF21* gene. By measuring the relative ASE within individuals, this approach has the ability to maximize detection of *cis*-regulatory variation on *TCF21* gene expression, with each allele controlled by similar *trans*-acting and environmental influences. Based on TaqMan SNP genotyping assays of total white blood cell RNA and genomic DNA from 22 heterozygous individuals (from GENEPAD cohort), we observed an approximate 1.3–2.0 fold ASE of the minor protective allele (G) over the major risk allele (C) in 18/22 samples, *P* = 1.1×10^−8^ (Fig. 1A). Importantly we observed consistent allelic imbalance (1.8–2.5 fold ratio G/C) in primary human coronary artery smooth muscle cells (HCASMC) maintained under normal conditions and detected using pyrosequencing assays (Fig. 1B). Together these data suggest that the disease-associated risk allele, or other variants in tight LD, contribute to decreased *TCF21* allele-specific expression. Intriguingly, these results contrast with published eQTL data at rs12190287, which demonstrate the risk allele is associated with elevated *TCF21* expression in omental adipose and liver tissues [2], [11]. Also, our recent work elucidated a bi-directional mechanism involving both *trans*-activating AP-1 and *trans*-repressing (Wilms tumor 1) WT1 transcription factor binding to a *cis*-regulatory element at rs12190287 resulting in altered allele-specific *TCF21* expression levels [10]. Given this bi-directional mode of transcriptional regulation we explored alternative regulatory mechanisms to potentially explain the allelic imbalance at rs12190287.

### Predicted miR-224 binding and altered *TCF21* 3′-UTR secondary structure at rs12190287

**Figure 2:**
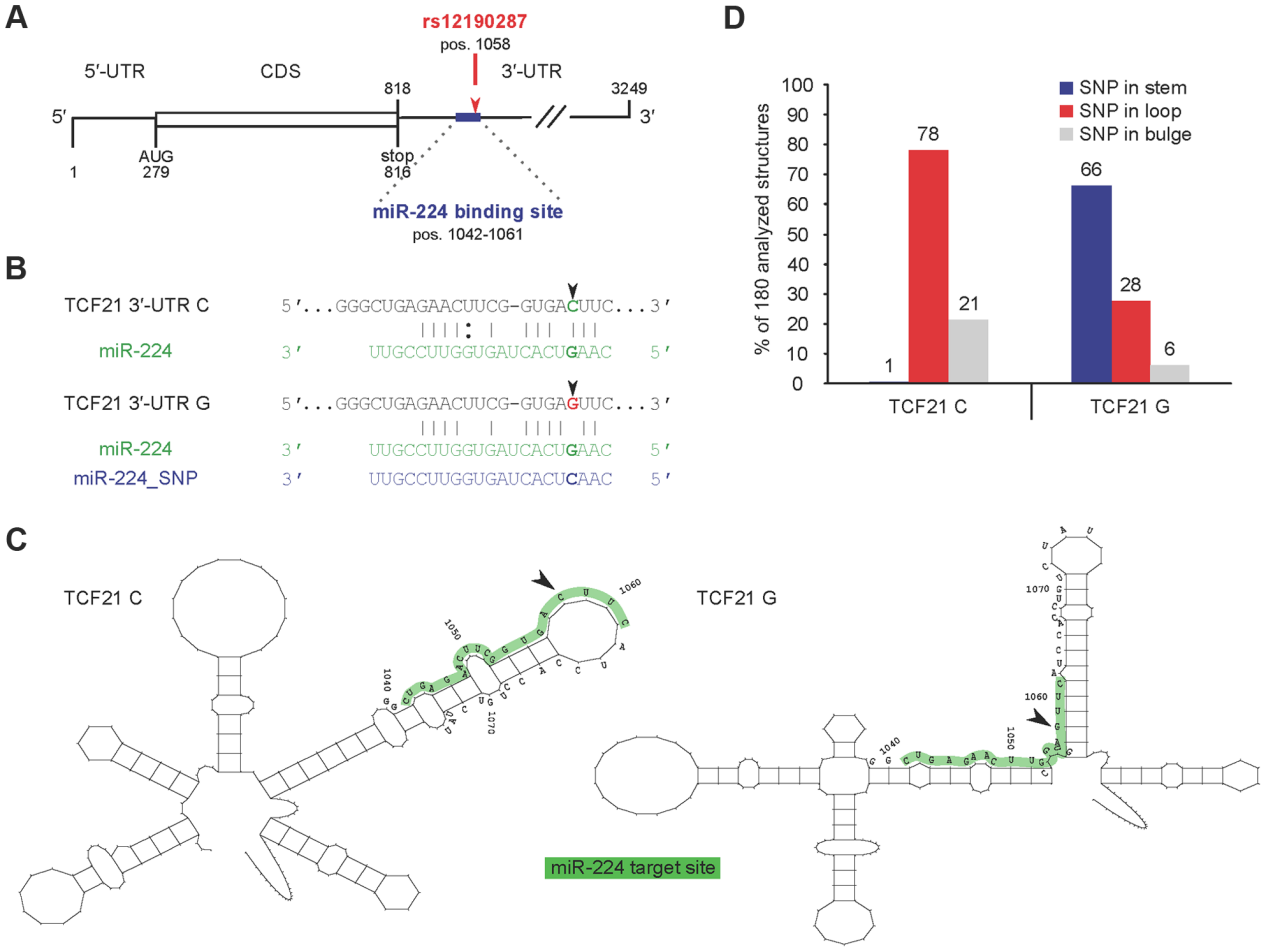
Predicted interaction of miR-224 with *TCF21* variant 1 3′-UTR and secondary structural changes at rs12190287. (**A**) TargetScan and MiRanda prediction algorithms identified rs12190287 (position 1058) residing within a 7-mer, mammalian conserved binding site for mature miR-224 (represented as an exact seed match for nucleotides 2–8; positions 1042–1061) (Fig. 2A). (**B**) Alignment of rs12190287 major and minor alleles demonstrated a perfect seed match between the *TCF21* 3′UTR containing the major risk allele (C) and miR-224, and a seed mismatch between the minor protective allele (G) and miR-224. (**C**) Systematic *in silico* RNA secondary structure predictions were performed as described in the text. Representative predicted structures of *TCF21* rs12190287 C and G variants are shown from positions 941–1141 (Fig. 2B). While both variants have similar global structures, they adopt distinct local secondary structures in proximity of the SNP. (**D**) Summary of SNP rs12190287 location within 180 analyzed RNA secondary structures, resulting in major risk allele (TCF21 C) typically located in loop region, while minor protective allele (TCF21 G) was often located in the stem region.

Recent studies using allelic imbalance sequencing demonstrate that SNPs frequently alter microRNA-mediated repression, by creating or disrupting complementary miRNA binding sites [12]. *In silico* analyses, based on conservation of miRNA seed regions, predict >60% of human 3′-UTRs are under selective control by miRNAs [13], [14]. We scanned the *TCF21* 3′UTR for seed matches using both TargetScan and MiRanda prediction algorithms. Both tools identified rs12190287 (position 1058 from 5′-UTR) residing within a 7-mer mammalian conserved binding site for mature miR-224 (Fig. 2A). Alignment of rs12190287 major and minor alleles demonstrated a perfect seed match between the *TCF21* 3′-UTR containing the major risk allele (C) and miR-224 (nucleotides 2–8; positions 1042–1061), with ΔΔG = −2.43 and a seed mismatch between the minor protective allele (G) and miR-224 (ΔΔG = 4.67) (Fig. 2B).

We also investigated the RNA secondary structure of the *TCF21* 3′-UTR variants. Systematic *in silico* RNA structural predictions were performed and analyzed as previously described [15], [16]. Representative predicted local secondary structures of *TCF21* rs12190287 C and G variants are shown from positions 941–1141 (Fig. 2B). While both 3′-UTR variants adopt similar global RNA structures, they are predicted to adopt distinct local secondary structures in proximity of the SNP. For instance, the seed matching sequence of the C variant seems to be mostly located within a loop structure and overall, the segment complementary to miR-224 (shaded grey) are located in a structurally accessible local structure (Fig. 2C). In contrast, the miR-224 binding sequence segment of the G variant is located in a local structure that does not seem to be accessible, i.e., the seed-matching element is located near a stem-loop junction within an intra-molecular duplex element (Fig. 2C). Theses observations were consistent among the 180 different structures analyzed, with the C variant SNP typically located in a loop structure and the G variant SNP often located along the stem (Fig. 2D). Similar differences in local RNA structure were predicted using the RNAfold minimal free energy (MFE) prediction algorithm (Supplementary Fig. S1). The overall difference in MFE for these structures is predicted to be only 2 kcal/mol, suggesting that the structure containing the C variant is slightly less stable. Different local RNA structures could also involve differential recruitment of RNA binding proteins (RBP), such as Pumilio, previously shown to alter p27 3′-UTR local structure and miR-221/222 accessibility [17]. In summary, these significant allelic structural differences implicate differences in miR-224 accessibility, binding kinetics, and binding affinity, which may impact miR-224 mediated regulation of *TCF21*.

### miR-224 dependent post-transcriptional regulation of *TCF21* 3′UTR at rs12190287

**Figure 3:**
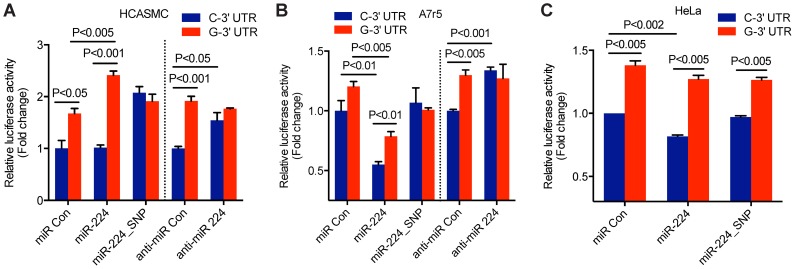
Allele-specific miR-224 regulation of *TCF21* 3′UTR at rs12190287. Luciferase reporter assay of *TCF21* rs12190287-C and G 3′-UTR variants determined in (**A**) primary coronary artery smooth muscle cells (HCASMC), (**B**) rat aortic smooth muscle cell line, A7r5, and (**C**) HeLa cell line. Negative control miRNA (miR Con), miR-224, miR-224_SNP, anti-miR negative control (anti-miR Con) or anti-miR-224 inhibitors were co-transfected with 3′-UTR reporters for 24 hrs and the relative luciferase activity (ratio of firefly/*Renilla* luciferase activity) was measured and normalized to C-3′-UTR+miR Con or anti-miR Con, shown as fold change. Data represent mean ± SEM of triplicates. Similar results were observed from three independent experiments. P-values are shown for intra and inter-assay comparisons where statistically significant (P<0.05).

We first evaluated the possibility that the *TCF21* 3′-UTR variants at rs12190287 differentially regulate protein expression through miR-224 targeting using a pmiR-GLO luciferase reporter system. The *TCF21* 3′-UTR variants (containing the major or minor alleles) were inserted downstream of the firefly luciferase gene, *luc2* to quantitatively measure post-transcriptional effects of miRNA activity, as previously described [18]. We synthesized miR-224 guide and passenger strands using miRBase sequences to generate double-stranded miR-224, which has a matched seed sequence of mature miR-224 to the C allele of the *TCF21* 3′-UTR target site but a mismatch to the G allele of *TCF21* 3′-UTR (Supplementary Fig. S2, top). In order to test the specificity of the miRNA-mediated regulation of the C variant we restored base-pairing in the seed region by synthesizing a miR-224 guide strand with a G>C substitution (referred to as miR-224_SNP, Supplementary Fig. S1, bottom). Using HCASMC co-transfected with the *TCF21* 3′-UTR reporters and double-stranded miR-224, we observed selective repression of the C variant compared to the G variant (Fig. 3A). Allele-specific differences in reporter activity were abolished when we co-expressed the adapting miR-224_SNP. Alternatively, using a loss-of-function approach with a selective miR-224 inhibitor, we observed increased reporter activity only by the C variant. These results further suggest that the C variant of the *TCF21* 3′-UTR can be directly regulated by miR-224, while the G variant cannot. We also observed similar functional effects in the aortic smooth muscle cell line A7r5 (Fig. 3B) and HeLa cells (Fig. 3C). However, the observation that miR-224_SNP did not completely block the allele-specific reporter activity in HeLa, may suggest cell type differences in endogenous miR-224 levels. For instance, both TCF21 and miR-224 are weakly expressed in A7r5 and HeLa cells relative to HCASMC (unpublished observations). Taken together, these data support a functional role of miR-224 in various cell types including HCASMC, by preferentially targeting the *TCF21* 3′UTR C variant, leading to post-transcriptional repression.

### Distinct *in vitro* annealing kinetics between miR-224 and *TCF21* 3′UTR variants

**Figure 4:**
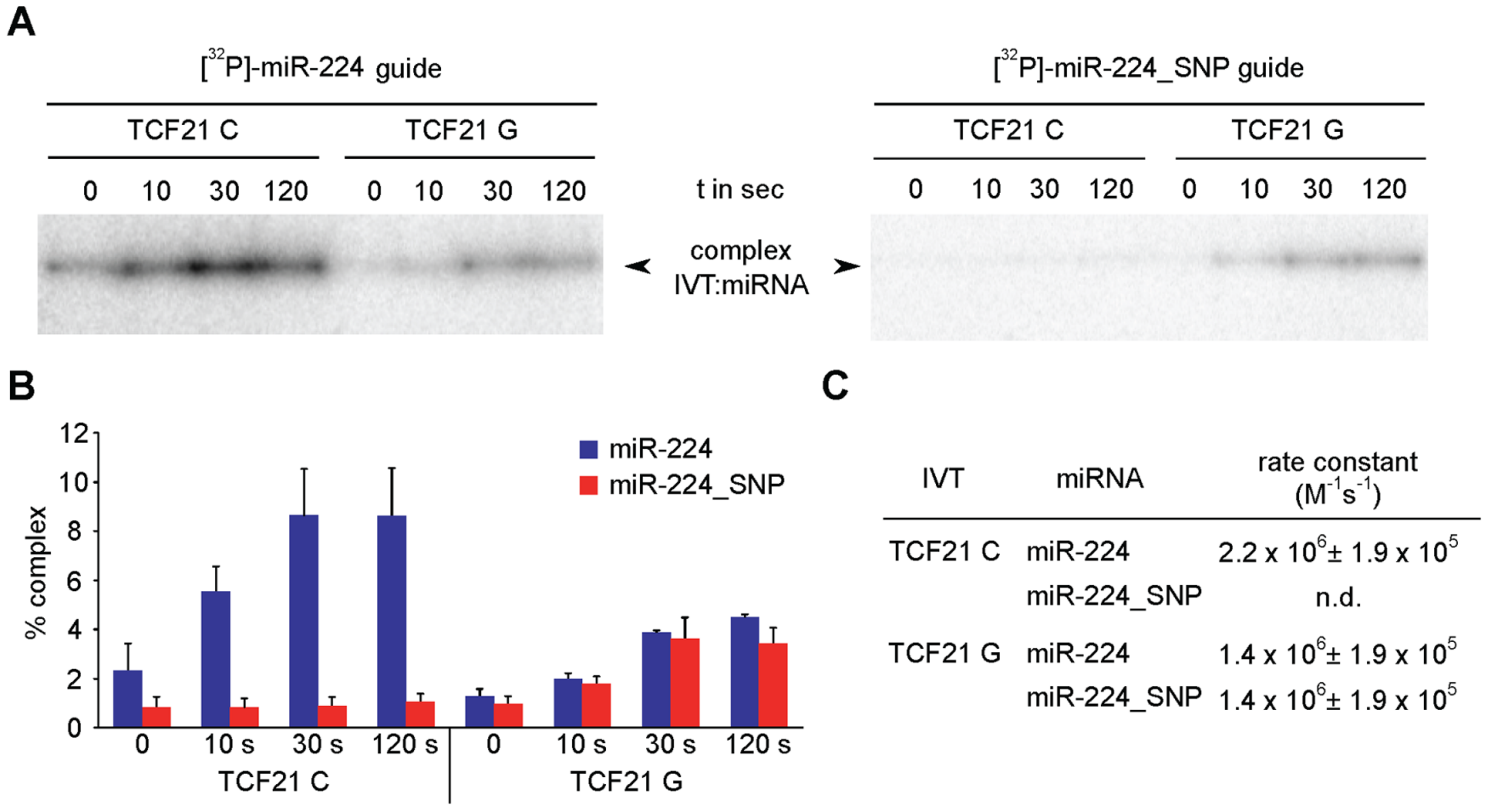
*In vitro* annealing kinetics between miR-224 and *TCF21* 3′UTR variants. (**A**) (Left panel) *TCF21* 3′-UTR variants were generated by *in vitro* transcription (IVT) and incubated with excess over ^32^P-labeled miR-224 for various time points, followed by autoradiography detection. Band intensities indicate relative amounts of the 3′-UTR variant:miRNA complexes formed over indicated times. (Right panel) IVT 3′-UTR variants were also incubated with excess over ^32^P-labeled miR-224_SNP, resulting in a seed mismatch with the C variant and a seed match with the G variant. (**B**) Band intensities of 3′-UTR:miRNA complexes formed using ^32^P-labeled miR-224 or ^32^P-labeled miR-224_SNP were detected by PhosphorImager and to quantify the percentage of complex signals ImageQuant-Software was used to determine relative to whole lane signal. Values represent mean ± SEM from three independent experiments. (**C**) Calculation of second-order rate constants for individual mRNA:miRNA complexes was performed as previously described [54]. n.d., complex formation was too slow to derive a rate constant.

A striking positive relationship exists between the extent of regulation and the annealing kinetics of the RNA regulator and its target RNA [19], [20]. Thus, differential regulation of *TCF21* 3′UTR variants by miR-224 could result from altered kinetics of mRNA:miRNA complex formation. We monitored the annealing kinetics of miR-224 binding to the *TCF21* 3′-UTR C and G variants *in vitro* under experimental conditions that are assumed to mimic known cellular facilitators of RNA:RNA annealing [21]. It is important to note that RNA:RNA annealing can be substantially promoted even at the cost of binding energy, i.e. the association of complementary ribonucleic acids can be greatly increased without lowering the Arrhenius activation energy or even significantly altering RNA structure [21]. The full-length *TCF21* 3′-UTR variants were generated by *in vitro* transcription (IVT) and incubated in 10-fold excess with ^32^P-labeled miR-224 for various time points, followed by autoradiography detection. Interestingly, greater amounts of the C variant 3′-UTR:miRNA complexes were generated over time compared with the G variant (Fig. 4A, left panel). Further, the C variant:miRNA complex formation was completely blocked in the presence of ^32^P-labeled miR-224_SNP, which generates a seed mismatch (Fig. 4A, right panel). However, the miR-224_SNP, which would generate a seed match with the G variant had no effects on G variant:miRNA complex formation. The C variant *TCF21* 3′-UTR:miR-224 complexes also formed at a faster rate (*k_obs_* = 2.2×10^6^ M^−1^ s^−1^) than the G variant (*k_obs_* = 1.4×10^6^ M^−1^ s^−1^) as determined from second-order reactions (Fig. 4B and 4C). Together these data suggest that miR-224 preferentially binds the major risk C variant of *TCF21*, and at a faster rate *in vitro*, compared to the minor protective G variant.

### Different structural conformations of *TCF21* 3′-UTR RNA determined by *in vitro* probing

**Figure 5:**
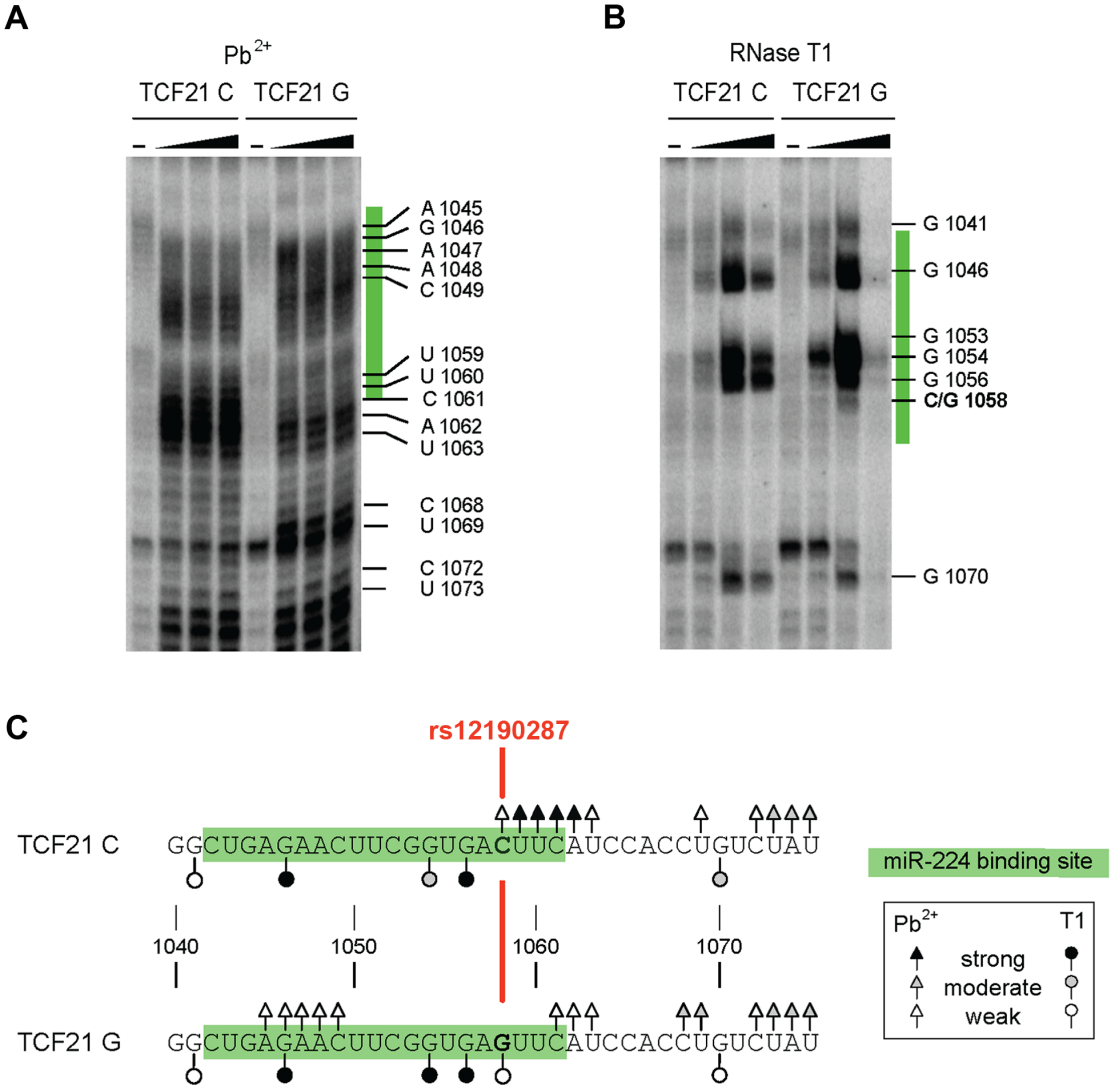
Allele-specific structural differences in conformations of *TCF21* 3′-UTR RNAs determined by *in vitro* probing. (**A**) Results of chemical probing of the *in vitro* transcribed *TCF21* 3′-UTR variants with varying amounts of Pb^2+^ (0, 10, 20 and 40 mM) and (**B**) enzymatic probing with varying amounts of RNase T1 (0, 0.25, 1 and 2 units). Major cleavage sites are shown along with their positions. miR-224 binding site is highlighted in green and rs12190287 is shown in red. (**C**) Summary of the major cleavage sites using either Pb^2+^ or RNase T1 and their overall cleavage strength is indicated by open and closed triangle and circles. Results are representative of at least three independent experiments.

We then used RNA *in vitro* probing to test and validate our *in silico* secondary structure predictions for the *TCF21* 3′-UTR variants, which demonstrated allele-specific local structural alterations. Briefly, we chemically probed the *TCF21* 3′-UTR variants with Pb^2+^ (to monitor all unpaired nucleotide residues) and probed enzymatically using RNase T1 (only cleaves unpaired G nucleotide residues). After probing, the cleavage patterns were evaluated by primer extension and subsequent denaturing gel electrophoresis, as described [18]. Probing the *TCF21* 3′-UTR variants (positions 1040–1075) with Pb^2+^ revealed unique cleavage sites proximal to the miR-224 target site (positions 1042–1061) and rs12190287 (position 1058) (Fig. 5A). For instance, the C variant has stronger and additional sites located proximal to rs12190287 (positions 1058–1063) in comparison to the G variant, which has some unique weak cleavage sites at positions 1045–1049. The specificity of RNase T1 to cleave G residues explains the occurrence of an additional weak cleavage product at the SNP position (1058) for the G but not C IVT (Fig. 5B). Additional stronger cleavage by RNase T1 was observed at position 1054 of the G variant, and a weaker cleavage at position 1070 of the G variant, summarized below (Fig. 5C). Together, these results are in line with the *in silico* predicted RNA structures, which imply there are a number of local structural differences between the two variants, resulting in altered accessibility at sites near rs12190287. It should be noted, however, that the structure-function relationship of RNA-RNA annealing is complex. Since the pairing mechanism of this *TCF21* case is not known, we cannot relate local structures, annealing kinetics, and biological effects. Nonetheless we observe differences at all levels of interaction, strongly suggesting a mechanistically distinct regulation.

### TGF-β and PDGF-BB signaling mediate inverse-correlated miR-224 and *TCF21* expression and ASE in HCASMC

**Figure 6:**
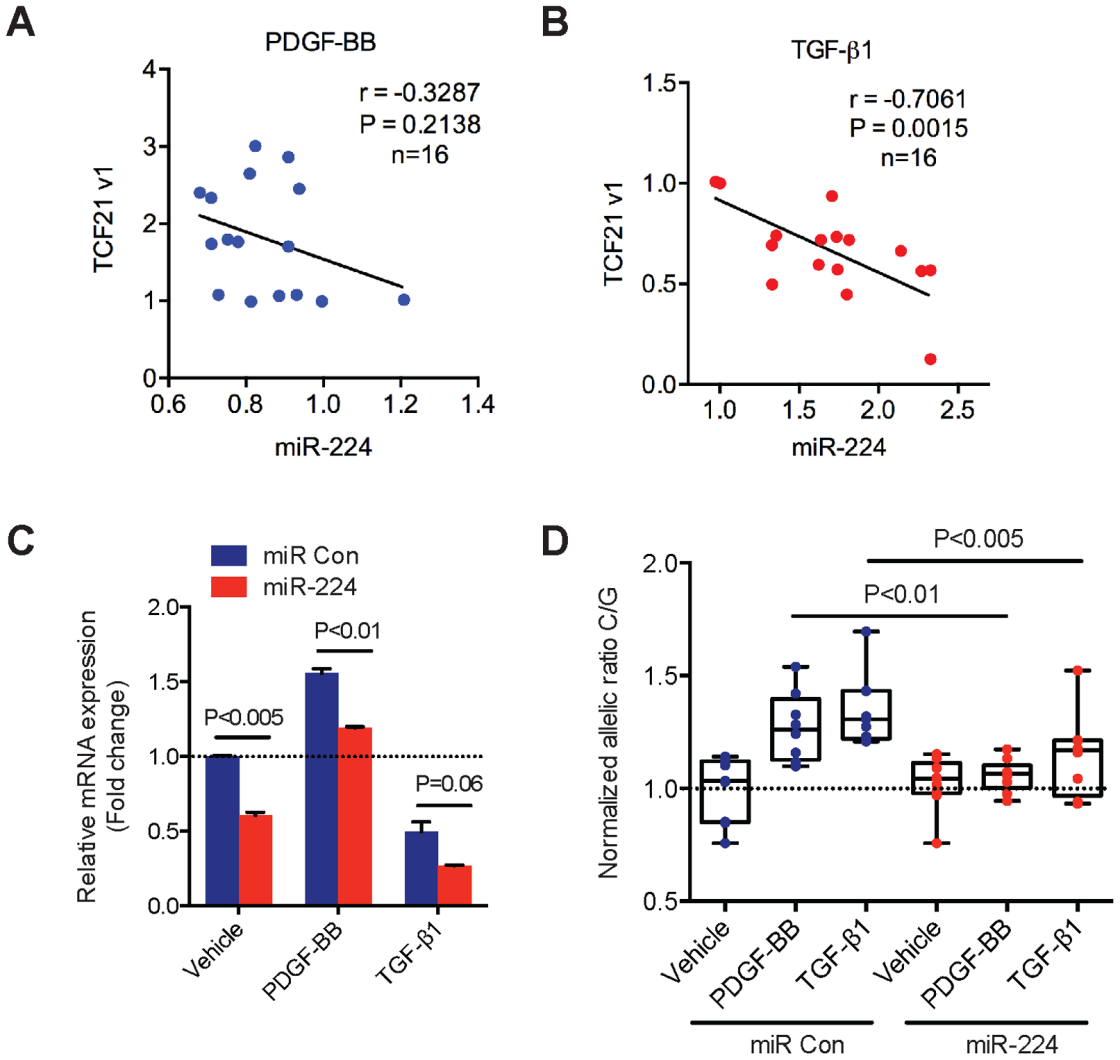
Correlation of endogenous *TCF21* and miR-224 expression levels in HCASMC. (**A**) TaqMan quantitative PCR results showing the correlation of endogenous *TCF21* variant 1 expression levels with miR-224 in HCASMCs stimulated with recombinant human PDGF-BB (20 ng/ml) for various time points (0, 1, 2, 6, 12, 24 hrs) (n = 16). (**B**) Similar experiments performed in HCASMCs stimulated with recombinant human TGF-β1 (5 ng/ml) for various time points (0, 1, 2, 6, 12, 24 hrs) (n = 16). *TCF21* and miR-224 expression levels were normalized to 18S and RNU44, respectively. Pearson's correlation was determined assuming a linear relationship, with resulting r, and P-values shown. (**C**) TaqMan qPCR results measuring total *TCF21* transcript levels in HCASMC transfected with Negative control miRNA mimic (miR Con) or miR-224 mimic and stimulated with either PDGF-BB or TGF-β1 for 6 hrs. Data represent mean ± SEM of triplicates. Similar results were observed from three independent experiments. (**D**) Allele-specific TaqMan qPCR measuring *TCF21* expression at rs12190287 in HCASMCs treated as described above. Values are expressed as the normalized ratio of C/G alleles. Data represent individual replicates from three independent experiments (n = 8–9).

Next, we investigated the regulatory pattern of endogenous *TCF21* and miR-224 gene expression levels in HCASMC. We first explored a potential link between relevant pathways upstream of miR-224 and *TCF21* that may account for miR-224-*TCF21* 3′-UTR allele-specific regulation in HCASMC. Importantly, our previous work identified platelet-derived growth factor (PDGF) and transforming growth factor-beta (TGF-β) dependent signaling pathways as respective positive and negative mediators of *cis*-regulatory elements at rs12190287 in HCASMC [10]. PDGF-BB ligand mediates increased SMC proliferation, survival, and migration [22] through PDGFRβ, which is critical for epithelial-mesenchymal transition (EMT) and formation of coronary artery SMC [23]. As a pleiotropic vasoactive cytokine, transforming growth factor beta (TGF-β1) also regulates EMT and diverse SMC growth and remodeling processes [24]. Interestingly, we observed a modest negative correlation (r = −0.3287) of endogenous *TCF21* and miR-224 expression levels in HCASMC treated with PDGF-BB, although this result did not reach statistical significance (Fig. 6A). However, TGF-β1 treatment resulted in significant and highly inverse-correlated endogenous *TCF21* and miR-224 expression levels, r = −0.7061, P = 0.0015 (Fig. 6B). We then measured the effects of these stimuli on miR-224-mediated regulation of total and allele-specific *TCF21* transcript levels. As expected, PDGF-BB treatment led to increased total *TCF21* expression levels, whereas TGF-β1 led to reduced *TCF21*, which was blunted in all cases by pre-miR-224 (Fig. 6C). We also observed pre-miR-224 to attenuate both PDGF-BB and TGF-β1 stimulated allele-specific *TCF21* expression (shown as the normalized ratio of C/G at rs12190287) (Fig. 6D). These results identify PDGF-BB and particularly TGF-β1 as potential upstream mediators of miR-224 directed allele-specific *TCF21* expression at rs12190287.

### TCF21 and miR-224 expression in human atherosclerotic coronary artery lesions

**Figure 7:**
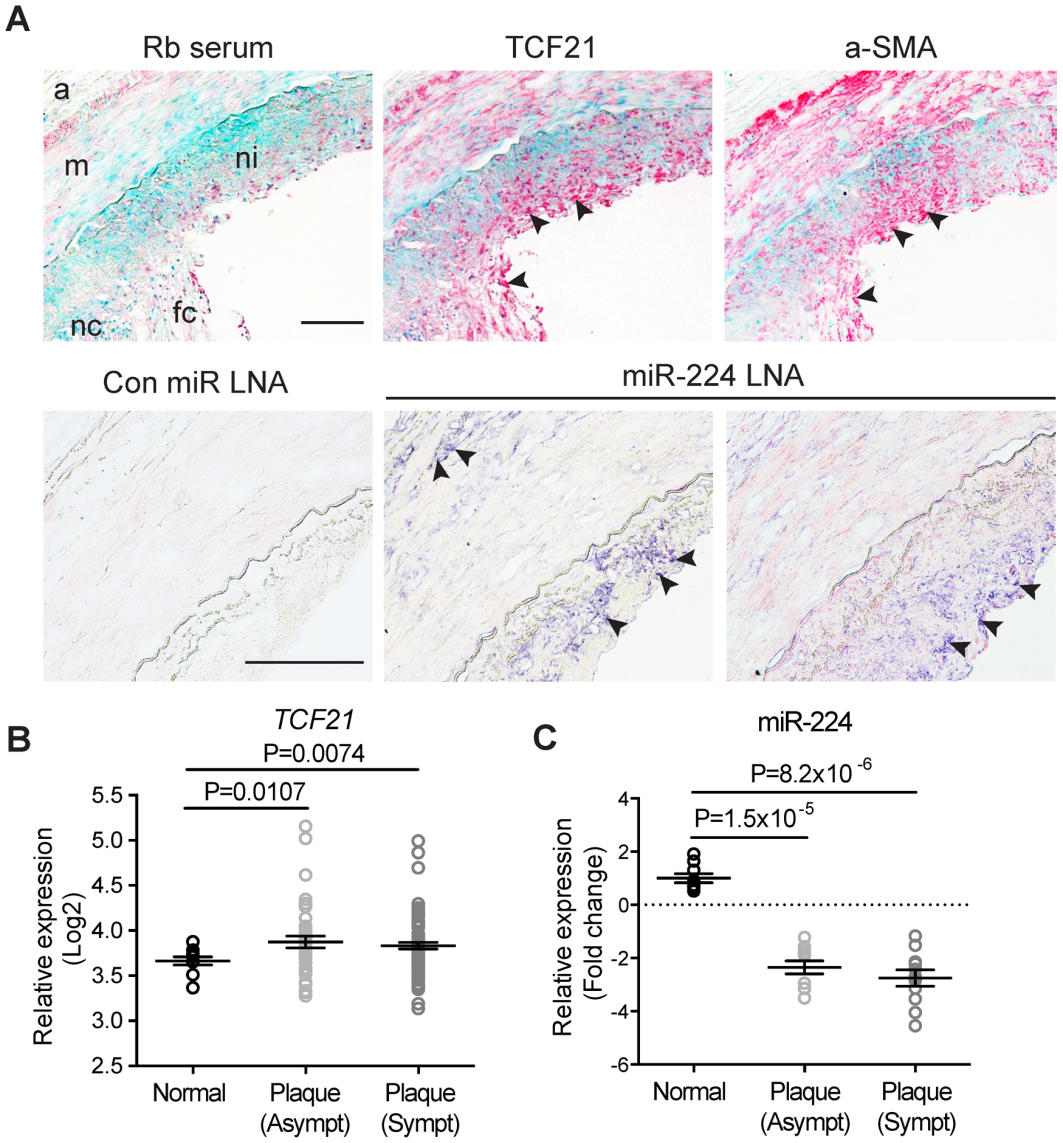
Expression of TCF21 and miR-224 in human atherosclerotic lesions. (**A**) (Upper panel) Immunohistochemical staining results showing endogenous TCF21 protein expression (in red) in neointima and media regions of left anterior descending (LAD) coronary artery sections (10× magnification). Adjacent sections were incubated with rabbit serum (negative control) or anti-alpha-smooth muscle actin (a-SMA) antibody to localize smooth muscle-like cells. Methyl Green was used as a nuclear counterstain. (Lower panel) Representative *in situ* hybridization results showing endogenous miR-224 (in indigo) localized in the neointima and adventitia in adjacent LAD sections (20× magnification). Rb: rabbit, LNA: locked nucleic acid. a: adventitia, m: media, ni: neointima, fc: fibrous cap, necrotic core. Arrows denote specific staining. Scale bars = 0.5 mm. (**B**) Microarray gene expression results showing regulation of *TCF21* mRNA and (**C**) TaqMan quantitative PCR results depicting miR-224 expression, during disease progression in normal, stable and unstable human carotid atherosclerotic lesions (n = 10 per group). Microarray-based expression levels were normalized by robust multi-array average (RMA) and TaqMan-based levels were normalized to the RNU44 internal control. Values represent mean Log2 fold change of replicates and lines represent mean ± SEM. Similar results were observed from two independent experiments.

To establish a potential role of miR-224-TCF21 regulation during atherosclerosis progression, we measured endogenous levels of miR-224 and TCF21 in human coronary artery lesions. Immunohistochemical staining of adjacent sections demonstrated TCF21 protein localized within the neointimal and medial layers of the left anterior descending (LAD) coronary artery (n = 4) (Fig. 7A, upper panel). TCF21 marked a population of cells resembling smooth muscle cells, indicated by alpha-smooth muscle actin (a-SMA) immunoreactivity in similar regions. TCF21 protein was also detected in the adventitia in a few samples, consistent with the expression pattern observed in small intramyocardial coronary arteries [25]. We also localized endogenous miR-224 in these sections using *in situ* hybridization, which identified miR-224 in both the neointimal and adventitial layers, but not the medial layer (Fig. 7A, lower panel). We validated these findings using microarray based analysis of normal (no lesions), stable (asymptomatic) and unstable (symptomatic) carotid atherosclerotic lesions. *TCF21* mRNA levels were significantly upregulated in both asymptomatic (P = 0.0106) and symptomatic (P = 0.0074) atherosclerotic plaques (Fig. 7B). Interestingly, miR-224 was significantly downregulated in stable and unstable atherosclerotic plaques (P = 1.5×10^−5^ and P = 8.2×10^−6^, respectively), as determined by TaqMan qPCR (Fig. 7C). These data confirm that both TCF21 protein and miR-224 are expressed in the diseased vessel wall *in vivo*, and their expression is inversely regulated during atherosclerosis, consistent with our observations in HCASMC. Together these findings provide additional mechanistic insights into the *TCF21* association with respect to coronary heart disease progression.

### Genome-wide prediction of disease-associated variants overlapping both TF and miRNA binding sites

It is also noteworthy that the minor protective allele at rs12190287 disrupts both a TF binding motif TGACTTCA as well as a miRNA seed sequence, GUGACUU in the 3′UTR of *TCF21*. Given this unanticipated integration of both positive *cis*-acting transcription factor binding and negative post-transcriptional miRNA regulation at *TCF21*, we sought to estimate the overall frequency of these overlapping regulatory features in humans using publicly available genome-wide datasets. Using validated TF binding ENCODE ChIP-seq regions (∼4,400,000) intersected with medium conserved miRcode predicted miRNA binding sites (∼1,100,000) we identified approximately 290,000 overlapping regions (approximately 28% of all predicted miRNA binding sites) (Supplementary Fig. S3). We then intersected total disease-associated polymorphisms from the National Human Genome Research Institute (NHGRI) catalog, including those in LD at r^2^>0.8 (∼292,000), resulting in 52,263 sites (17.9%) in TF ChIP regions, 942 sites (0.32%) in miRNA binding sites, and 146 sites overlapping with both features (0.05%) (Fig. S3A and Table S1). Interestingly, this overlap was less frequent when applied to all common variants (12.8%, 0.16%, and 0.04%, respectively) (Supplementary Fig. S3B). We also observed 20,064 (37%) regions of TargetScan predicted conserved miRNA binding sites residing within TF ChIP-seq peaks. Functional annotation of these regions resulted in significant enrichment of mitogen activated protein kinase (MAPK) (P = 1×10^−39^), cytokines (P = 1×10^−54^), and TGF-β (P = 1×10^−23^) pathways (Supplementary Fig. S3D), as well as bZip (P = 1×10^−81^) and p53 (P = 1×10^−29^) TF binding protein domains versus those expected by chance (Supplementary Fig. S3C). Given the critical role of bZip domain TF families (e.g. AP-1, ATF and CREB) in various cancers, inflammation and developmental processes [26], concurrent miRNA binding to mRNA regions overlapping these sites (e.g. miR-224-*TCF21*) may represent an exquisite fine-tuning control of target gene expression.

## Discussion

A large fraction of CHD susceptibility loci recently identified through GWAS do not appear to be mediating risk through effects on traditional risk factors, such as lipid levels and blood pressure. Investigating the mechanism(s) of the disease risk association at these loci promises to provide critical new information regarding fundamental disease pathways in the vessel wall that function upstream of the causal variation and the related causal gene [1], [2], [27]. One gene that we have chosen to study in this regard is *TCF21*, a gene that was originally identified and replicated in the CARDIoGRAM meta-analysis of GWA data, and has now been verified through additional meta-analysis in both subjects of European and Han Chinese descent [1]–[3]. *TCF21* encodes a basic-helix-loop-helix transcription factor that is involved in controlling cell fate decisions in developing coronary artery SMC, and may provide insight into the possible causal role of this cell type in atherosclerosis [8], [9].

Initial eQTL analysis showed that *TCF21* expression is related to the genotype at rs12190287, providing the first suggestion that *TCF21* is indeed the causal gene at this locus (Table S3) [2]. These data, in conjunction with evidence that rs12190287, (1) is associated with a *P*-value that is three orders of magnitude lower than that for other associated SNPs within the susceptibility locus [2], (2) is only modestly correlated with other SNPs that reach genome-wide significance within the locus [10], (3) is found in a region of open chromatin configuration [10], and (4) resides within the *TCF21* structural gene, collectively suggest that rs12190287 is the causal variant within this susceptibility locus. To further investigate this possibility, we have pursued allele-specific expression (ASE) studies as reported here, seeking to correlate ASE with genotype at rs12190287. These studies employing RNA from circulating leukocytes show highly significant ASE at the *TCF21* gene, and consistent allelic expression divergence suggests that rs12190287 is the causal SNP. Although possible, it would seem very unlikely that the ASE is due to another SNP in high linkage disequilibrium with this SNP, since other associated variants are correlated at best with an r^2^∼0.6. Also, while leukocytes are an appropriate cell type in atherosclerosis and express a number of the signaling components upstream of *TCF21*, they may not be the primary cell type reflecting TCF21 function. In this regard, we observed a consistent direction of ASE in a limited study of primary cultured HCASMC grown in the presence of serum. Unfortunately, eQTL studies with circulating leukocytes did not show a significant association with *TCF21* expression so we could not compare the directionality between the leukocytes and the adipose and liver tissues employed in the original eQTL studies.

miRNAs predominately affect gene expression by decreasing mRNA stability or inhibiting translation. These regulatory effects can be perturbed by allelic variation through SNPs directly interfering with basepair interactions in the seed sequence in the mRNA. Allelic variants can also alter the tertiary structure of the mRNA and hinder miRNA binding even when the SNP is located outside the seed sequence [28], [29]. Here, we employed reporter gene studies in HeLa cells, rat and human SMCs with both gain and loss of function approaches to demonstrate that miR-224 regulates TCF21 expression at the protein level. Sequence analysis predicts that rs12190287 alters the core miR-224 binding sequence, and folding algorithms that identify lowest energy conformations of the native and variant sequences suggest that the minor G allele at rs12190287 produces a less favorable configuration of mRNA folding for miR-224 binding. These hypotheses were confirmed by kinetic studies showing decreased rate and extent of miR-224 binding, and RNA structural probing studies that revealed decreased availability of the miR-224 binding region in the mRNA containing the minor G allele. The disruption of miRNA binding is a well-established mechanism for alteration of risk for various cancers [30]. However, these data showing that the CHD causal variant rs12190287 can disrupt miR-224 binding provides the first evidence for this type of mechanism for coronary disease associated genes.

While a potential role for miR-224 in regulating vascular disease has not been defined, this miRNA has been studied in association with multiple cancer cell types and other cellular systems, and these data provide some insight into upstream pathways that might affect *TCF21* expression and thus CHD risk [24], [31]–[36]. Most significant among these are NFκB, WNT and TGF-β, all of which have been linked to atherosclerotic signaling pathways [24], [34], [36]. NFκB is a well-characterized transcription factor and mediator of cellular activation by inflammatory cytokines and chemokines, and in the context of hepatocellular carcinoma, miR-224 was shown to be upregulated by tumor necrosis-α (TNF-α) and miR-224 regulation linked to hepatocellular migration and invasion [31]. TGF-β stimulation of miR-224 expression has been characterized in ovarian granulosa cells where it has been implicated in cellular proliferation and estradiol release in this cell type [32]. Further, miR-224 has been shown to be upregulated by the WNT signaling pathway in meduloblastoma where it was linked to inhibition of proliferation, increased radiation sensitivity and reduced anchorage-independent growth of tumor cells [35]. Each of these pathways has been linked to atherosclerotic processes in the diseased blood vessel wall, and could have a role in the *TCF21* mediated risk for CHD [24], [33], [34], [36].

**Figure 8:**
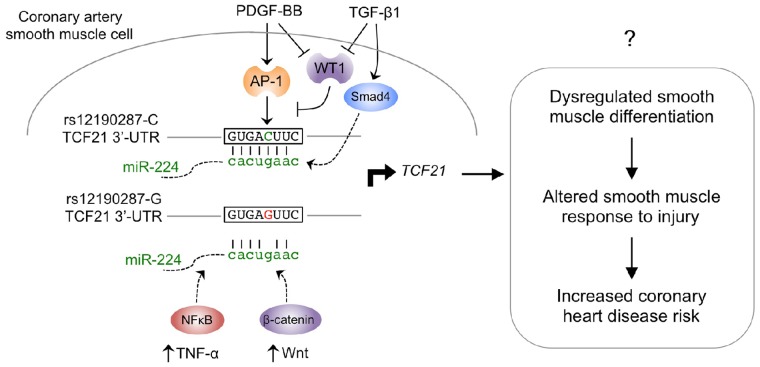
Proposed model of signaling pathways converging on miR-224-*TCF21* interaction at rs12190287. Previous studies elucidated a *cis*-regulatory mechanism by which the lead SNP associated with CHD at 6q23.2, rs12190287, was shown to disrupt trans-activating AP-1 binding by the minor protective allele (G). This resulted in altered growth factor mediated transcriptional activation, chromatin organization and allele-specific *TCF21* gene expression in human coronary artery smooth muscle cells (HCASMC). The *trans*-repressing factor, Wilms tumor 1 (WT1) was also shown to counter-regulate the positive effects of AP-1 at rs12190287 and preferentially associate with the major risk allele (C). Herein we describe a post-transcriptional *cis*-regulatory mechanism by which the minor protective allele alters a perfect seed match of miR-224 in the 3′-UTR of *TCF21*. Altered RNA structure is predicted to account for differential miRNA binding kinetics, and regulation of transcription. Further, both PDGF-BB and TGF-β upstream stimuli in HCASMC may account for the miR-224 mediated allele-specific expression at rs12190287. Additionally, both NFκB and Wnt upstream signals have been proposed to regulate miR-224 in tumor cells, and may potentially participate in the described mechanisms in HCASMC. Taken, together dysregulation of *TCF21* is predicted to account for altered smooth muscle cell (SMC) response to injury due to phenotypic modulation from a differentiated to proliferating SMC, leading to increased risk for CHD.

Merging these data with that from previous studies of the transcriptional regulation at rs12190287 provides a more complete picture of the complexity of upstream signaling pathways that may regulate *TCF21* expression, and may be perturbed by this disease-associated variant. We have shown that rs12190287 resides in an atypical AP-1-like element and that PDGF can stimulate allele-specific expression through this site, as one potential disease-related pathway activated at this region [10]. PDGF has been extensively implicated in atherosclerosis pathogenesis, and *in vitro* genomic studies have suggested that TCF21 mediates PDGF signaling ([37], and data not shown). Additionally, transcriptional regulation studies at rs12190287 have also shown that the Wilms tumor factor (WT1) inhibits expression of *TCF21* through the AP-1-like site, and PDGF and TGF-β stimulation shown to be upstream inhibitors of WT1 expression in SMC [10]. WT1 is known to inhibit expression of AP-1 like factors, and has been shown to repress *TCF21* expression in developmental models [38]–[40]. Combining these data with that derived here for miR-224 provides compelling evidence for multiple signaling pathways, operating by transcriptional and post-transcriptional mechanisms, by which rs12190287 regulates *TCF21* expression (Fig. 8). Importantly, this is the first example of a disease-associated variant that disrupts both transcription factor-DNA and miRNA-mRNA interactions. Our genome-wide analysis provides further support that additional disease-associated variants reside in overlapping TF and miRNA binding regions, which likely have pathophysiological relevance (Supplementary Fig. S3). We can speculate that this bimodal regulation may partially explain the “dynamic” eQTLs previously observed [41], [42] which are responsive to intracellular changes in differentiation state.

Previous work from this laboratory has characterized a transcriptional regulatory mechanism that mediates *TCF21* gene expression differences through variation at rs12190287, and studies presented here documents a second mechanism by which variation at this SNP can alter expression of individual alleles [10]. Inherent in such mechanistic studies at disease-associated loci is that altered allele-specific expression can alter disease risk through either, 1) changing the overall causal gene expression level to alter the normal biological role of the causal gene or to introduce a novel disease-promoting function; or 2) changing the overall variance of causal gene expression, such that the gene becomes disconnected from its normal signaling networks [43]. The former may be explained by increased transcription of the rs12190287 risk “C” allele resulting in overall increased *TCF21* expression, or the miRNA mechanism by which the “C” allele interacts with miR-224 to decrease overall *TCF21* expression. It is possible that these counteracting pathways trigger overall *TCF21* expression variance linked to a disease-related phenotype, with different pathways being dominant in different disease contexts. Evidence for directionality of *TCF21* expression is provided here in the context of carotid artery disease, with atherosclerotic vessels showing increased *TCF21* expression (Fig. 7). These data are consistent with eQTL data in adipose and liver tissue samples which indicated that the risk “C” allele at rs12190287 is associated with increased *TCF21* expression, suggesting that the transcriptional mechanism at rs12190287 may play a dominant role on gene expression levels (Table S3). In addition, we also demonstrate that miR-224 is reciprocally decreased in these carotid diseased tissues, suggesting that miR-224 may function as a repressor of aberrantly elevated *TCF21* levels, but is blunted in the process. It is important to consider that transcription factor and miR-224 pathways are regulated by multiple upstream pathways, which can regulate expression and/or activation of TFs and miR-224. Verification of the direction of effect for *TCF21* expression, and the mechanisms that function at rs12190287 will require additional studies with human vascular disease samples to better assess *in vivo* gene expression in the disease environment. Also, studies in Tcf21 genetic mouse models should provide additional evidence of the direction, and mechanism of effect, in the setting of vascular disease. It is well known that *TCF21* is protective for multiple human cancers and it will be of great interest to determine whether expression of this gene in disease-related cells inhibits or promotes vascular disease processes [44]–[47].

Finally, to fully understand the complexity of transcriptional and miRNA networks regulating *TCF21* expression, it will be essential to investigate the risk contributed by additional alleles associated with disease at this locus. GWAS of an East Asian cohort identified the CHD associated variant rs12524865 in the *TCF21* locus, and the fact that this SNP is poorly correlated with rs12190287 in this racial ethnic group suggest that it is an independent allele [3]. Previous studies from this laboratory suggested that this variant may directly regulate *TCF21* expression through transcriptional pathways similar to those associated with rs12190287 [10]. In addition, fine-mapping studies by the CARDIoGRAM+C4D consortium has identified a second associated allele in Caucasians centered around a variant ∼100,000 basepairs upstream of *TCF21*, rs17062853, with this variant being poorly correlated with rs12190287 in Caucasians, again suggesting this is an independent allele [1]. It will be important to investigate these alleles independently and collectively to begin to understand how they contribute to *TCF21* regulation in the context of smooth muscle biology and disease processes. To better assess the pathophysiological role of different alleles it will be essential to conduct ASE analyses in cells isolated from human vascular lesions. These future studies may reveal how allelic variation at *TCF21* affects relevant upstream signaling pathways during different disease states.

## Materials and Methods

### Allelic expression imbalance using TaqMan quantitative PCR

Peripheral blood DNA and RNA were isolated from randomly selected buffy coat samples from individuals of European descent in two human cohort studies, GENEPAD (Genetic determinants of Peripheral Artery Disease) and GENESiPS (GENEticS of insulin sensitivity iPSc). Genomic DNA was isolated using the Qiagen DNeasy Blood and Tissue kit according to the manufacturer's instructions. Genotypes at rs12190287 were determined from 10 ng gDNA template using a predesigned TaqMan SNP genotyping assay for rs12190287 (Applied Biosystems) and performed in triplicate. Sanger sequencing also confirmed heterozygous samples at rs12192087. Total RNA was isolated using the Qiagen miRNeasy Mini kit according to the manufacturer's instructions. Total cDNA was prepared from 1 µg RNA using the High Capacity cDNA Reverse Transcription kit (Applied Biosystems, #4368814). cDNA templates were used to amplify allele-specific *TCF21* using the TaqMan SNP genotyping probe (Applied Biosystems). ASE was determined from 22 heterozygous samples using the TaqMan SNP genotyping probe for rs12190287 and expressed as the normalized allelic ratio of cDNA/gDNA. Calibration of the SNP genotyping assay was determined as previously described [10].

### Allelic expression imbalance using pyrosequencing [10]

DNA and RNA were prepared from 8 individual HCASMC lots determined to be heterozygous at rs12192087 (confirmed by Sanger sequencing). Pyrosequencing assays for rs12190287 were performed as previously described with assays designed using PyroMark Assay Design software (Qiagen). Forward rs12190287 PCR primer, 5′-biotinylated reverse PCR primer, and forward pyrosequencing primers (Table S2) were synthesized by the Protein And Nucleic acid (PAN) facility (Stanford). Approximately 20 ng gDNA or cDNA was amplified using forward and reverse pyrosequencing primers under the following conditions: 94°C 4 min, (94°C 30 s, 60°C 30 s, 72°C 45 s) ×45, 72°C 6 min. PCR products were verified by gel electrophoresis. Pyrosequencing reaction was performed on PCR reactions using a PyroMark Q24 according to manufacturer's instructions. Allelic quantitation was obtained automatically from the mean allele frequencies derived from the peak heights using PyroMark Q24 software.

### Cell culture [10]

Primary human coronary artery smooth muscle cells (HCASMC) were purchased from three different manufacturers, Lonza, PromoCell and Cell Applications and were cultured in complete smooth muscle basal media (Lonza, #CC-3182) according to the manufacturer's instructions. All experiments were performed with HCASMC between passages 4–7. Genotypes of HCASMC were determined as described above, and lots heterozygous at rs12190287 were used for all experiments. The A7r5 rat aortic SMC line was purchased from ATCC and cells were maintained in Dulbecco's modified Eagle medium (DMEM, Life Technologies, #11885-084) containing low glucose, sodium pyruvate and L-glutamine and supplemented with 10% fetal bovine serum (FBS). HeLa cells were maintained in DMEM containing high glucose, sodium pyruvate and L-glutamine supplemented with 10% FBS.

### Dual-luciferase assay

Double stranded DNA sequences containing the *TCF21* 3′-UTR for rs12190287-C and G were subcloned into the multiple cloning site (MCS) of the pmirGLO vector (Promega, #E1330), located downstream of the translation stop codon and firefly luciferase reporter gene *luc2*, driven by the PGK minimal promoter and also carrying the renilla luciferase reporter gene *hRluc*, as an internal control. PCR and mutagenic primer sequences to generate the *TCF21* C and G 3′-UTR reporters are included in Table S2. Site-directed mutagenesis protocol was adapted from [48]. For gain-of-function studies, single-stranded, unmodified oligonucleotides for miR-224 (seed-matching TCF21 C allele) and miR-224_SNP (seed-matching *TCF21* G allele) were first annealed at an equimolar concentration at 95°C for 3 min and allowed to gradually cool to room temperature. Resulting double-stranded miR-224, miR-224_SNP or negative control miRNAs (Ambion/Life Technologies) were co-transfected at 50 nmol/L along with *TCF21* C-3′-UTR or G-3′UTR reporter constructs in HeLa, HCASMC or A7r5 using Lipofectamine 2000 (Invitrogen/Life Technologies, #11668-019) according to the manufacturer's instructions. Alternatively, loss-of-function studies were carried out by co-transfecting 50 nmol/L anti-miR-224 or negative control anti-miR inhibitors (Ambion/Life Technologies). Culture media was changed after 6 hrs, and dual luciferase activity was measured after 24 hrs using either SpectraMax L luminometer (Molecular Devices) or anthos Lucy3 luminometer (anthos Mikrosysteme GmbH). Relative luciferase activity (firefly/*Renilla* luciferase ratio) is represented as the fold change of respective control condition as indicated.

### Quantitative miRNA, total mRNA and allele-specific gene expression

HCASMC were maintained as described above under normal growth factor and serum supplemented conditions. Upon reaching ∼70% confluence cells were serum-starved overnight prior to stimulation with human recombinant PDGF-BB or TGF-β1 for various times in triplicates. Samples were randomized (n = 16) and total RNA was isolated using the miRNeasy Mini kit (Qiagen). Total cDNA was prepared from 1 µg RNA using the High Capacity cDNA Reverse Transcription kit (Applied Biosystems, #4368814). Alternatively, miRNA specific cDNA was prepared using the TaqMan miRNA Reverse Transcription kit (Applied Biosystems/Life Technologies, #4366596) and predesigned RT probes for human miR-224 or human control miRNA RNU44 (Applied Biosystems/Life Technologies). cDNA templates were used to measure endogenous human miR-224 and *TCF21* variant 1 (TCF21 v1) expression levels using predesigned TaqMan gene expression assay probes (Applied Biosystems/Life Technologies) according to the manufacturer's instructions. *TCF21* v1 and miR-224 levels were quantitated on a ViiA 7 Real-Time PCR system (Applied Biosystems) and normalized to 18S and RNU44 levels, respectively. Pearson's correlation was determined assuming a linear relationship.

For expression analyses with miR-224 overexpression, HCASMC were cultured as described above under normal conditions. The day after plating the cells were transfected with either miR negative control (miR Con) or miR-224 mimic using Lipofectamine RNAiMAX (Life Technologies, #13778150) for 5 hrs. Culture media was changed to serum-free and cells were incubated overnight prior to stimulation for 6 hrs with either vehicle, 20 ng/ml human recombinant PDGF-BB (R&D Systems, #220-BB-010), or 5 ng/ml human recombinant TGF-β1 (R&D Systems, #240-B-002). Total RNA was isolated using the RNeasy isolation kit (Qiagen, #217004) and cDNA was prepared as described above. Total *TCF21* or allele-specific expression at rs12192087 was measured as described above using the TaqMan gene expression or TaqMan SNP genotyping probe with expression levels calculated using a standard curve and normalized to the gDNA for each allele.

### Determination of annealing rate constants for complementary RNA [18]

Observed association rate constants (*k*_obs_) were measured as previously described in detail [49], [50]. Briefly, 5′ radioactively labeled miR-224 or miR-224_SNP (0.5 nM final concentration) was incubated with the *TCF21* 3′-UTR-C or *TCF21* 3′-UTR-G target mRNA at 5 nM final concentration in hybridization buffer (100 mM NaCl, 20 mM Tris–HCl, pH 7.4, and 10 mM MgCl_2_) in the presence of 10 mM CTAB at 37°C. Aliquots were withdrawn at different time points, transferred into 1 vol of stop buffer (20 mM Tris–HCl, pH 7.4, 10 mM EDTA, 2% (v/v) SDS, 8 M urea, 0.025% (v/v) bromophenol blue) and analyzed by native polyacrylamide gel electrophoresis (0,1×11×12 cm, run at 4°C and 150 V for 2 h). Gels were sealed in polyethylene; exposed to X-ray film stored at −20°C until band intensities were determined using a phosphorimager (Typhoon 8600 Variable Mode Imager, GE Healthcare). ImageQuant 5.2-software was used to quantify signals relative to whole lane signal. Second order association rate constants were calculated as described [20].

### Probing and primer extension [18]

PCR products harboring the RNA polymerase T7 recognition site were amplified for *TCF21* with 5′-GAA ATT AAT ACG ACT CAC TAT AGG GCC TTG GAG TTT GGT ACC TGG-3′ as forward, 5′- TCA GGT CGA CTT GGT GGA ACA AAT CTT TTA TTT TC-3′ as reverse primer and the pmirGLO *TCF21* 3′-UTR constructs as template and used for *in vitro* transcription (T7 RiboMAX Express Large Scale RNA Production System, Promega, #P1320). *In vitro* transcripts (IVTs) were purified by phenol-chloroform-extraction, G-50 column filtration and ethanol precipitation, and subsequently denatured for 10 min at 70°C and refolded at room temperature for 120 min. RNase T1 based hydrolysis: IVTs were incubated at RT for 4 min in 10 µl reaction containing 1 µg tRNA (Sigma, #83853-25MG) and increasing RNase T1 (0, 0.25, 1 and 2 units, Fermentas, #EN0541). Cleavage products were purified as described above. Pb^2+^ based probing: refolded IVTs were incubated for 15 min at RT in a 10 µl reaction mix. Reactions were initiated with 5 µg tRNA and increasing amounts of lead(II) acetate (Pb^2+^) (Sigma, #32307), terminated after 10 min at RT with EDTA/ethanol, followed by ethanol precipitation. RT reactions with either RNase T1- or lead hydrolysis products were performed for 45 min at 42°C using 1 mM dNTPs, 2.5 mM RT-primer (5′-^32^[P] -AGG GCA TCC TGA CAT CTT GA-3′) and 1.5 units AMV Reverse Transcriptase (Promega, #M5108). Sequencing reactions were performed in parallel with denatured IVT (2 min at 95°C) for each nucleotide base, as adapted from [49]. After cDNA synthesis, samples were denatured in formamide-containing loading buffer for 3 min at 95°C and resolved on a 10% polyacrylamide sequencing gel under denaturing conditions for 70 min at 52°, and signals analyzed with a PhosphorImager (Typhoon 8600 Variable Mode Imager, GE Healthcare).

### miRNA annealing [18]

Single-stranded miRNA guide and passenger strands (miR-224 and miR-224_SNP, Fig. 2B; miR-224 guide: 5′-CAA GUC ACU AGU GGU UCC GUU-3′, miR-224_SNP guide: 5′-CAA CUC ACU AGU GGU UCC GUU-3′ and miR-224 passenger: 5′- AAA AUG GUG CCC UAG UGA CUA CA -3′) were synthesized by biomers.net GmbH. Double-stranded miRNA was generated by incubating the two strands at a final concentration of 20 µM in 1× RNA annealing buffer (6 mM Tris-HCl pH 7.4, 20 mM KCl, 0.4 mM MgCl_2_). The annealing reaction was performed by denaturing the oligonucleotides (3 min at 95°C) and subsequent slow cooling in a heat block. The hybridization product was analyzed by native PAGE.

### Computational analysis of RNA secondary structure [18]

*In silico* folding of RNA sequences was performed using an adaptation of the mfold package [51], [52] that has been modified to work with the Accelrys Genetics Computer Group. The calculations were performed with the polymorphic sequence segments containing the SNP at varying internal positions and by defining stepwise (10–25 nt) moving segments with sizes of 100, 200, 400 and 800 nt. The resulting structures were compared globally and locally at the SNP position and/or the respective miRNA-binding site and grouped according to the involvement of the SNP-containing sequence segment in intramolecular folding. We validated our predicted structures with the RNAfold package (University of Vienna) using minimum free energy (MFE) based structure calculations from varying length segments containing the SNP.

### *In silico* transcription factor and miRNA binding intersection and enrichment analysis

Genome-wide binding regions for hg19 ENCODE transcription factor ChIP V3 and miRcode V11 or TargetScan miRNA binding sites were extracted using the Galaxy tool. Resulting bed files were intersected with latest GWAS SNP catalog (in European populations) from the National Human Genome Research Institute (NHGRI), augmented with SNPs in LD at r^2^>0.8, to identify overlapping positions. Overlapping genomic regions of transcription factor binding and TargetScan miRNA binding sites were imported into the Genomic Regions Enrichment of Annotations Tool (GREAT) for functional assignment by pathway and motif analyses. Statistical enrichments were performed for associations between the overlapping genomic regions and the annotations using the whole genome as a background region.

### Immunohistochemistry

Major coronary arteries were dissected from explanted hearts of patients undergoing heart transplant at Stanford, as previously described [53]. Briefly, left anterior descending (LAD), circumflex, and right coronary arteries were dissected and macroscopically scored as disease (containing lesion) or normal (lesion-free), rinsed in saline and fixed in 4% paraformaldehyde overnight at 4°C, followed by cryopreservation in 10%, 20%, and 30% sucrose at 4°C for 30 min, 1 hr, and 2 hrs, respectively. Coronary segments were embedded in OCT media prior to sectioning at 7 µm thickness. Frozen slides were thawed and immunohistochemistry procedure was performed according to the manufacturer's protocol (Biocare Medical, #RMR625). Briefly, tissue sections were blocked for 30 min using a universal blocking reagent and endogenous peroxidases were quenched prior to incubation with rabbit anti-TCF21 (Abcam, #ab49475), mouse anti-ACTA2 (α-SMA; Sigma, #SAB1403519) primary antibodies or rabbit serum as a negative control (purified rabbit or mouse IgG were also used as negative control antibodies). Sections were washed in tris buffered saline (TBS) and incubated in respective alkaline phosphatase (AP) conjugated polymers for 30 min followed by detection using Vulcan Fast Red chromogen (Biocare Medical, #FR805). Nuclei were counterstained using Methyl Green (Vector Labs, #H3402). Images were captured on a Zeiss light microscope and total brightness and contrast were uniformly adjusted for each condition.

### *In situ* hybridization

Unlabeled miR-224 locked nucleic acid (LNA) and scrambled LNA control oligo probes were purchased from Exiqon and 100 pmol oligos were labeled using the digoxigenin (DIG) Oligonucleotide Tailing Kit, 2^nd^ generation (Roche, #3-353-583) according to the manufacturer's instructions. Labeled probes were purified using Sephadex G25 columns (GE Biosciences, #27-5325-01) according to the manufacturer's instructions and labeling efficiency was measured via dot blot analysis using serial dilutions of labeled LNA oligo and Control DIG-dUTP/dATP tailed oligo with detection using an anti-DIG-AP conjugated antibody (Roche, #1093274) and NBT/BCIP developer (Roche, #11697471001). Probes were diluted in hybridization buffer to a final concentration of 25 or 50 nM and linearized for 5 min at 65°C. Probes were added to thawed slides and incubated at 55°C in a humidified chamber for 2 hrs. Slides were washed with 5X, 1X, 0.2X SSC buffer for 15, 30, 15 min respectively, followed by 15 min wash in phosphate buffered saline (PBS). Slides were incubated in blocking solution containing 5% heat-inactivated sheep serum, 1% bovine serum albumin, 0.1% Tween-20 in RNase-free PBS. Slides were then incubated with AP-conjugated anti-DIG Fab fragment antibody (1∶1500, Roche, #1093274) for 2.5 hrs at RT. Slides were washed for 2×30 min in PBS-Tween 0.1% and 2×20 min in PBS. Signal was detected by incubating with NBT/BCIP developer with 1 mM Levamisole (Sigma) for 36–48 hr at RT in the dark. Nuclei were counterstained with Nuclear Fast Red (Vector) for 5 min, washed in running H2O and slides coverslipped with aqua-poly/mount (Polysciences). Images were obtained at 20× magnification using a light microscope.

### Microarray and TaqMan based gene expression in human atherosclerotic carotid arteries

Human atherosclerotic carotid artery lesions were obtained from patients undergoing endarterectomy surgery for stable (asymptomatic) (n = 40) or unstable (symptomatic) (n = 87) carotid stenosis, as part of the Biobank of Karolinska Endarterectomies (BiKE). Normal control arterial samples (n = 10) were obtained from the iliac and radial arteries from healthy organ donors without any history of cardiovascular disease. Briefly, tissue was snap frozen in liquid nitrogen before pulverizing to a fine powder using a pre-chilled mortar and pestle, then resuspended in Qiazol lysis reagent (Qiagen) and homogenized with a rotor stator tissue homogenizer. Total RNA was extracted as described above using the miRNeasy Mini Kit (Qiagen) and RNA quality assessed using a Bioanalyzer 2100 (Agilent). Global gene expression profiles were analyzed by Affymetrix HG-U133 plus 2.0 Genechip microarrays from 127 patient derived plaque samples and 10 donor control samples. Robust multi-array average (RMA) normalization was performed and processed gene expression data presented in Log2 scale. For TaqMan based analysis, miRNA-specific cDNA was prepared as described above, and TaqMan qPCR was performed in triplicates using predesigned TaqMan probes for miR-224 and normalized to the RNU44 internal control. Data are represented as mean Log2 fold change of replicates from two independent experiments.

### Statistical analysis

Experiments were performed using at least three independent preparations with individual treatments/conditions performed in triplicate [10]. Data is presented as mean ± standard error mean (SEM) of replicates. GraphPad Prism 6.0 was used for statistical analysis. For all *in vitro* comparisons between two groups, paired two-tailed *t*-test was performed. For carotid artery expression analyses between normal donor and endarterectomy plaque samples, unpaired two-tailed *t*-test with Welch's correction was performed. *P* values<0.05 were considered statistically significant. For multiple comparison testing, two-way analysis of variance (ANOVA) accompanied by Tukey's post-hoc test were used as appropriate.

### Ethics statement

All samples reported in this study were obtained with approval of the Institutional Review Board at Stanford University and under written informed consent from patients undergoing orthotopic heart transplantation (coronary arteries from explanted hearts), or those participating in the Genetic Determinants of Peripheral Artery Disease (GENEPAD) and Genetics of Insulin Sensitivity iPSC (GENESiPS) studies (peripheral blood). All atherosclerotic carotid plaque and donor control samples collected from the Biobank of Karolinska Endarterectomies (BiKE) were obtained with informed consent from patients, organ donors or their guardians. The BiKE study is approved by the Ethical Committee of Northern Stockholm.

## Supporting Information

Figure S1 Predicted minimal free energy based RNA structure of major and minor alleles of *TCF21* 3′-UTR using the RNAfold algorithm. Arrow and circle denotes location of rs12190287. Grey shaded bases highlight miR-224 seed region. Heat map represents base-pair probability for paired regions and unpaired probability for unpaired regions.
(TIF)

Figure S2 (Top) Alignment of endogenous miR-224 with major and minor alleles of *TCF21* 3′-UTR demonstrating a seed match and seed mismatch, respectively. (Bottom) Alignment of artificial miR-224 (miR-224_SNP) with major and minor alleles of *TCF21* 3′-UTR forming a seed mismatch and seed match, respectively.
(TIF)

Figure S3 Genome-wide overlap of ENCODE transcription factor ChIP binding regions and miRcode predicted miRNA binding sites (medium conserved) with (**A**) GWAS SNPs or (**B**) common SNPs (MAF>1%). Note: Venn diagrams are not to scale. (**C**) MSigDB and PANTHER pathway enrichment analysis of ENCODE transcription factor ChIP binding regions and highly conserved TargetScan predicted miRNA binding sites using GREAT. (**D**) MSigDB Promoter transcription factor motif, transcription factor DNA binding domain (InterPro) and MSigDB miRNA binding motif enrichment analysis of transcription factor ChIP regions and highly conserved TargetScan predicted miRNA binding sites using GREAT. Binomial p-values are shown, with the whole genome used as a background dataset.
(TIF)

Table S1 Results of intersected genome-wide transcription factor binding with medium conserved miRcode predicted miRNA binding sites and GWAS SNPs.
(XLS)

Table S2 Oligonucleotide sequences used in various assays.
(DOC)

Table S3 Summary of expression quantitative trait loci identified at 6q23.2. All expression associations with P<10^−5^ are shown where the coronary artery disease associated SNP is the strongest expression SNP (eSNP) in the region or is in high linkage disequilibrium (r^2^≥0.6) with the strongest SNP. ^1^Details of the tissue sources and analysis are reported in Schunkert H et al. 2011. ^2^Direction of effect for the associated eSNP. In all cases the major risk alleles were associated with higher gene expression (+), while the minor alleles were associated with lower gene expression. n.s. not significant.
(DOC)
