# Supplementary figures and images for: Coronary Heart Disease-Associated Variation in TCF21 Disrupts a miR-224 Binding Site and miRNA-Mediated Regulation
Source: PLoS Genet. 2014 Mar 27;10(3):e1004263. doi: 10.1371/journal.pgen.1004263 (PMC3967965; doi:10.1371/journal.pgen.1004263)

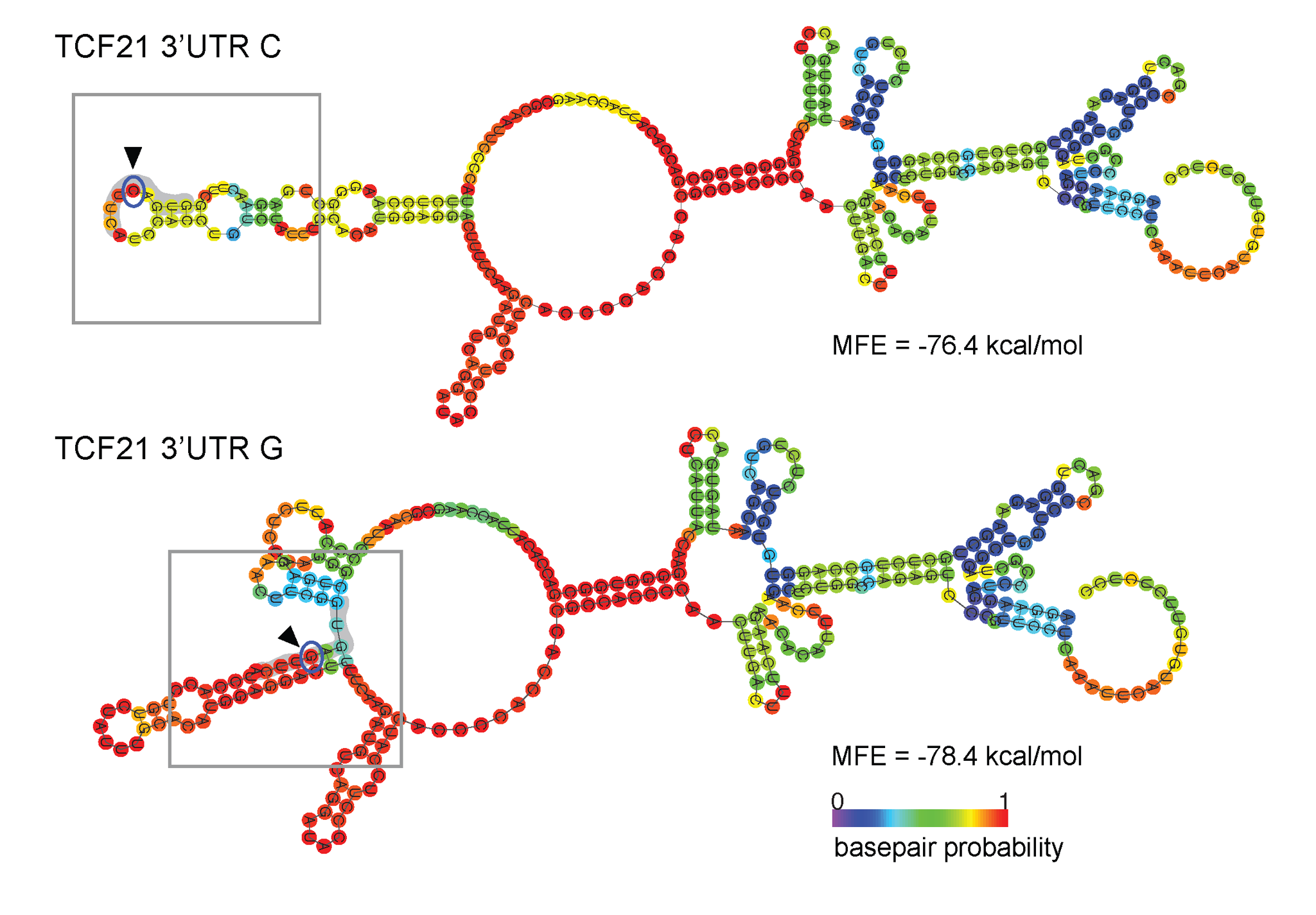

Supplement: Figure S1 — Predicted minimal free energy based RNA structure of major and minor alleles of TCF21 3′-UTR using the RNAfold algorithm. Arrow and circle denotes location of rs12190287. Grey shaded bases highlight miR-224 seed region. Heat map represents base-pair probability for paired regions and unpaired probability for unpaired regions. (TIF) [file pgen.1004263.s001.tif]

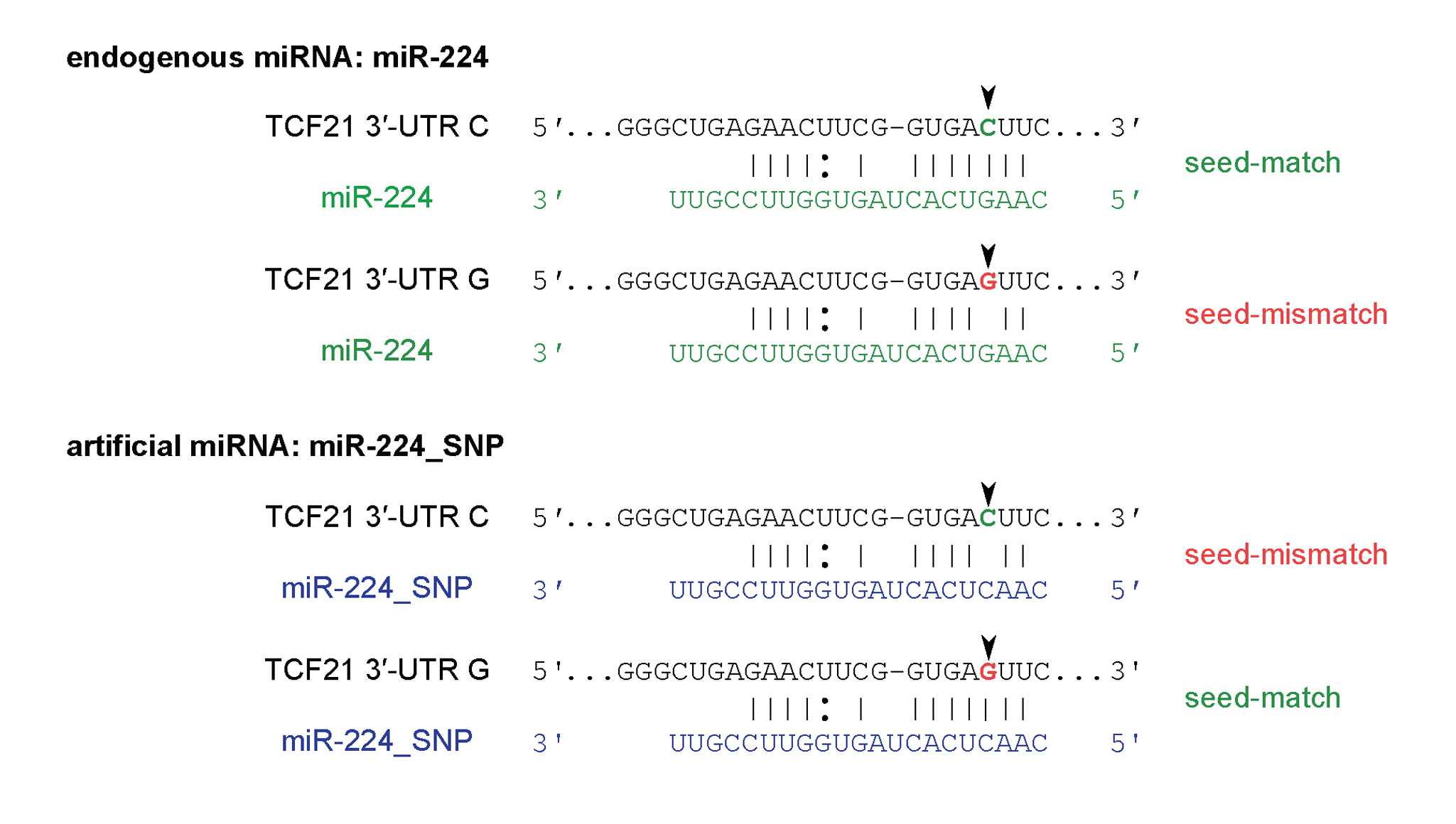

Supplement: Figure S2 — (Top) Alignment of endogenous miR-224 with major and minor alleles of TCF21 3′-UTR demonstrating a seed match and seed mismatch, respectively. (Bottom) Alignment of artificial miR-224 (miR-224_SNP) with major and minor alleles of TCF21 3′-UTR forming a seed mismatch and seed match, respectively. (TIF) [file pgen.1004263.s002.tif]

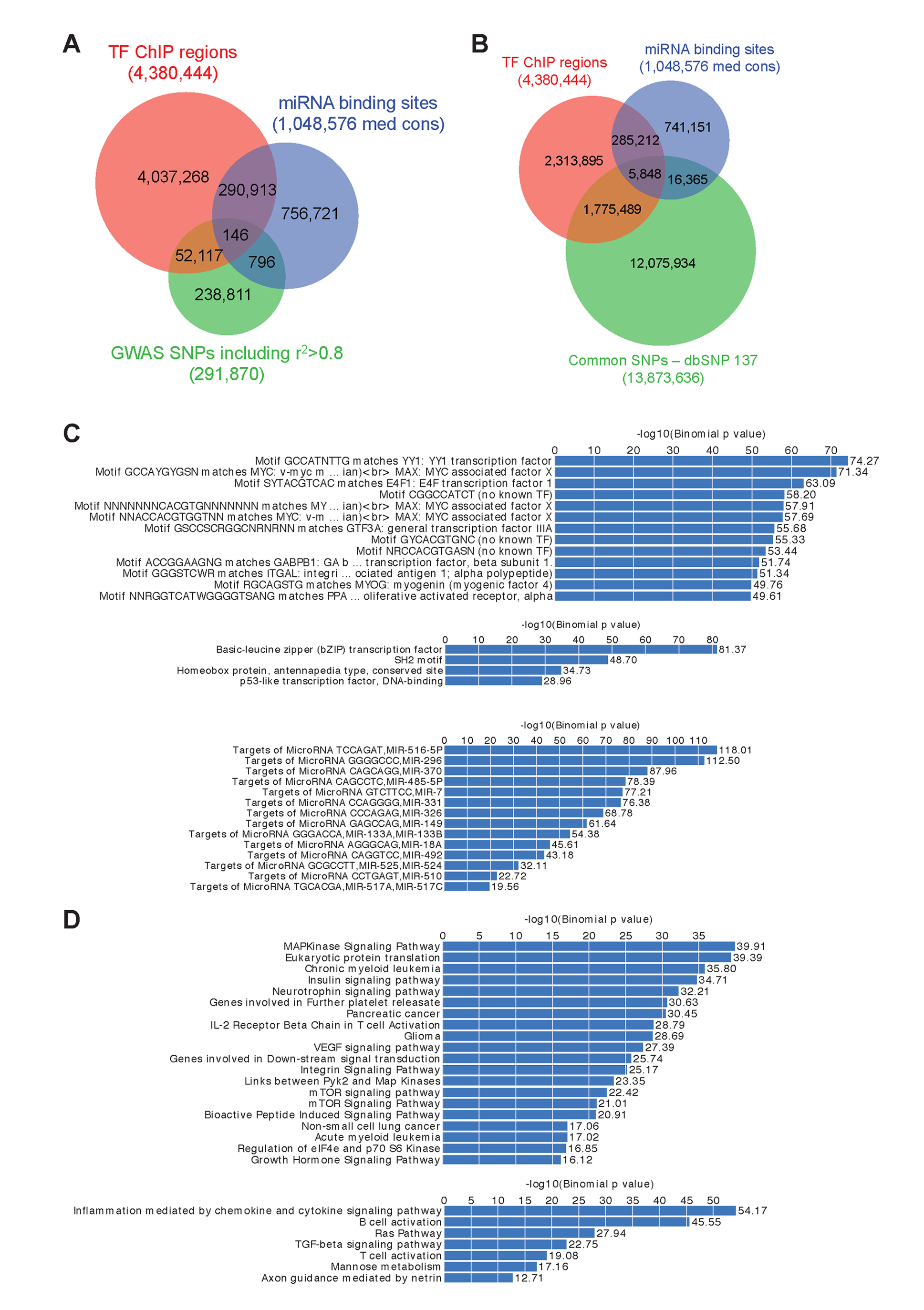

Supplement: Figure S3 — Genome-wide overlap of ENCODE transcription factor ChIP binding regions and miRcode predicted miRNA binding sites (medium conserved) with (A) GWAS SNPs or (B) common SNPs (MAF>1%). Note: Venn diagrams are not to scale. (C) MSigDB and PANTHER pathway enrichment analysis of ENCODE transcription factor ChIP binding regions and highly conserved TargetScan predicted miRNA binding sites using GREAT. (D) MSigDB Promoter transcription factor motif, transcription factor DNA binding domain (InterPro) and MSigDB miRNA binding motif enrichment analysis of transcription factor ChIP regions and highly conserved TargetScan predicted miRNA binding sites using GREAT. Binomial p-values are shown, with the whole genome used as a background dataset. (TIF) [file pgen.1004263.s003.tif]
