# Supplementary material for: Coronary Heart Disease-Associated Variation in TCF21 Disrupts a miR-224 Binding Site and miRNA-Mediated Regulation
Source: PLoS Genet. 2014 Mar 27;10(3):e1004263. doi: 10.1371/journal.pgen.1004263 (PMC3967965; doi:10.1371/journal.pgen.1004263)
Supplement: Table S2 — Oligonucleotide sequences used in various assays. (DOC) [file pgen.1004263.s005.doc]

Table S2. Oligonucleotide sequences

| **Assay** | **Oligonucleotide** | **Sequence (5’-3’) or Assay** |
| --- | --- | --- |
| Luciferase | TCF21 C 3’UTR For | AGAGTTTAAACCTTGGAGTTTGGTACCTGG |
|  | TCF21 C 3’UTR Rev | TCAGGTCGACTTGGTGGAACAAATCTTTTATTTTC |
|  | TCF21 G 3’UTR mut | CGGTGAGTTCATCCACC |
|  | miR-224 guide | CAAGUCACUAGUGGUUCCGUU |
|  | miR-224_SNP guide | CAACUCACUAGUGGUUCCGUU |
|  | miR-224 passenger | AAAAUGGUGCCCUAGUGACUACA |
| Probing | TCF21_IVT For | GAAATTAATACGACTCACTATAGGGCCTTGGAGTTTGGTACCTGG |
|  | TCF21_IVT Rev | TCAGGTCGACTTGGTGGAACAAATCTTTTATTTTC |
|  | TCF21_SNP RT | AGGGCATCCTGACATCTTGA |
| Genotyping | rs12190287 PCR For | GCTCTCTGTCAGCAATAGTGACCT |
|  | rs12190287 PCR Rev | TGTGTTCTTGAAAGTCAAGTTGGG |
|  | rs12190287 Seq For | ACCACATTACCAAGCGCAATTCCC |
|  | rs12190287 Seq Rev | ATGGAAGGGTATCCTGACATCTTG |
|  | rs12190287 PCR 1 For | GCTCTCTGTCAGCAATAGTGACCT |
|  | rs12190287 PCR 1 Rev | TGTGTTCTTGAAAGTCAAGTTGGG |
|  | rs12190287 Seq 1 For | ACCACATTACCAAGCGCAATTCCC |
|  | rs12190287 Seq 1 Rev | ATGGAAGGGTATCCTGACATCTTG |
|  | rs12190287 PCR 2 For | AGGCAGATCCTGGCTAACGACAAA |
|  | rs12190287 PCR 2 Rev | TGAATTTGAGGAACGGGACTCTGG |
|  | rs12190287 Seq 2 For | TGACCTTGGAGTTTGGTACCTGGA |
| Pyroseq | rs12190287 Pyro 1 For | Biotin-CCACATTACCAAGCGCAATT |
|  | rs12190287 Pyro 1 Rev | TGGGGTGATGGAAGGGTAT |
|  | rs12190287 Pyro Seq | ATAGACAGGTGGATGA |
| TaqMan | rs12190287 | C_32243431_10 |
|  | Human TCF21_1/2 | Hs00162646_m1 |
|  | Human TCF21_2 | Hs04230774_s1 |
|  | Human TCF21_1 | Hs01546814_m1 |
|  | Human 18S | Hs99999901_s1 |
|  | Human miR-224-5p | Hsa-miR-224 |
|  | Human Con miRNA | RNU44 |
