# Supplementary material for: Coronary Heart Disease-Associated Variation in TCF21 Disrupts a miR-224 Binding Site and miRNA-Mediated Regulation
Source: PLoS Genet. 2014 Mar 27;10(3):e1004263. doi: 10.1371/journal.pgen.1004263 (PMC3967965; doi:10.1371/journal.pgen.1004263)
Supplement: Table S3 — Summary of expression quantitative trait loci identified at 6q23.2. All expression associations with P<10−5 are shown where the coronary artery disease associated SNP is the strongest expression SNP (eSNP) in the region or is in high linkage disequilibrium (r2≥0.6) with the strongest SNP. 1Details of the tissue sources and analysis are reported in Schunkert H et al. 2011. 2Direction of effect for the associated eSNP. In all cases the major risk alleles were associated with higher gene expression (+), while the minor alleles were associated with lower gene expression. n.s. not significant. (DOC) [file pgen.1004263.s006.doc]

**Table S3. Summary of expression quantitative trait loci identified at 6q23.2.**

| **Band** | **SNP (risk allele)** | **Position (hg19)** | **Gene** | **Tissue**^1^ | **Effect**^2^ | ***P-*value** |
| --- | --- | --- | --- | --- | --- | --- |
| 6q23.2 | rs12190287 [C] | 134256218 | *TCF21* | Omental adipose | + | 1.20x10^-8^ |
| “ | “ | “ | “ | Liver | + | 2.30x10^-8^ |
| “ | “ | “ | *“* | Circulating leukocytes |  | n.s. |
| “ | rs12524865 [C] | 134196674 | *“* | Omental adipose | + | 3.78x10^-10^ |
| “ | “ | “ | “ | Liver | + | 1.41x10^-6^ |
| “ | “ | “ | *“* | Omental adipose | + | 1.88x10^-9^ |
| “ | “ | “ | “ | Liver | + | 3.27x10^-7^ |
| “ | “ | “ | *“* | Circulating leukocytes |  | n.s. |
| “ | rs1967917 [G] | 134198175 | *“* | Omental adipose | + | 3.72x10^-10^ |
| “ | “ | “ | “ | Liver | + | 1.40x10^-6^ |

Summary of expression quantitative trait loci (eQTL) identified at 6q23.2. All expression associations with P<10^-5^ are

shown where the coronary artery disease associated SNP is the strongest expression SNP (eSNP) in the region or is in high linkage disequilibrium (r^2^≥0.6) with the strongest SNP. ^1^Details of the tissue sources and analysis are reported in Schunkert H et al. 2011. ^2^Direction of effect for the associated eSNP. In all cases the major risk alleles were associated with higher gene expression (+), while the minor alleles were associated with lower gene expression. n.s. not significant.
